# Digital wound monitoring with artificial intelligence to prioritise surgical wounds in cardiac surgery patients for priority or standard review: protocol for a randomised feasibility trial (WISDOM)

**DOI:** 10.1136/bmjopen-2024-086486

**Published:** 2024-09-17

**Authors:** Judith Tanner, Melissa Rochon, Roy Harris, Jacqueline Beckhelling, James Jurkiewicz, Lara Mason, Janet Bouttell, Sarah Bolton, Jon Dummer, Keith Wilson, Luxmi Dhoonmoon, Karen Cariaga

**Affiliations:** 1 School of Health Sciences, University of Nottingham, Nottingham, UK; 2 Infection Prevention and Control, Guy's and St Thomas' NHS Foundation Trust, London, UK; 3 NIHR Research Support Service, Nottingham, UK; 4 University Hospitals of Derby and Burton NHS Foundation Trust, Derby, UK; 5 Isla Health, London, UK; 6 Centre for Healthcare Equipment and Technology Adoption, Nottingham, UK; 7 Health Innovation East Midlands, Nottingham, UK; 8 Liverpool Heart and Chest Hospital NHS Foundation Trust, Liverpool, UK; 9 Central and North West London NHS Foundation Trust, London, UK

**Keywords:** WOUND MANAGEMENT, Artificial Intelligence, Telemedicine, Clinical Trial, eHealth, Infection control

## Abstract

**Introduction:**

Digital surgical wound monitoring for patients at home is becoming an increasingly common method of wound follow-up. This regular monitoring improves patient outcomes by detecting wound complications early and enabling treatment to start before complications worsen. However, reviewing the digital data creates a new and additional workload for staff. The aim of this study is to assess a surgical wound monitoring platform that uses artificial intelligence to assist clinicians to review patients’ wound images by prioritising concerning images for urgent review. This will manage staff time more effectively.

**Methods and analysis:**

This is a feasibility study for a new artificial intelligence module with 120 cardiac surgery patients at two centres serving a range of patient ethnicities and urban, rural and coastal locations. Each patient will be randomly allocated using a 1:1 ratio with mixed block sizes to receive the platform with the new detection and prioritising module (for up to 30 days after surgery) plus standard postoperative wound care or standard postoperative wound care only. Assessment is through surveys, interviews, phone calls and platform review at 30 days and through medical notes review and patient phone calls at 60 days. Outcomes will assess safety, acceptability, feasibility and health economic endpoints. The decision to proceed to a definitive trial will be based on prespecified progression criteria.

**Ethics and dissemination:**

Permission to conduct the study was granted by the North of Scotland Research Ethics Committee 1 (24/NS0005) and the MHRA (CI/2024/0004/GB). The results of this Wound Imaging Software Digital platfOrM (WISDOM) study will be reported in peer-reviewed open-access journals and shared with participants and stakeholders.

**Trial registration numbers:**

ISRCTN16900119 and NCT06475703.

**STRENGTHS AND LIMITATIONS OF THIS STUDY**

- Mixed methods, including qualitative and quantitative data, will provide in-depth information on satisfaction and acceptability.
- The study will evaluate the feasibility, acceptability and safety of the intervention and provide data to inform an adequately powered subsequent randomised trial.
- The economic investigation comprises an initial scoping exercise and an economic evaluation on completion of the clinical study.
- A study is limited to testing feasibility and is not powered to test the effectiveness of the intervention.

## Introduction

 Surgical site infections (SSIs) are among the most common healthcare-associated infections globally, with the WHO reporting one-third of all patients in lower-income and middle-income countries developing a wound infection after surgery.^1^ These problematic and infected wounds are distressing for patients and can result in increased readmissions and further surgery.^2 3^ There are also additional costs to healthcare providers and to patients.^4 5^

Most wound problems, present after patients have been discharged from the hospital^6^ and this figure continues to increase through initiatives such as enhanced recovery programmes which promote early patient discharge.^7^ It is important to identify and treat problematic wounds quickly as delays in treating a wound can allow problems to worsen making them harder and more expensive to treat.^8^ Therefore, identifying wound problems and infections after discharge is becoming increasingly important.

Digital remote surgical wound monitoring offers a solution to monitoring wounds in patients’ own homes.^9^ With digital monitoring, patients upload images of their wounds and some information using their smartphone in response to weekly preprogrammed text messages which are then reviewed by specialist nurses.^10^

Evaluations of digital wound monitoring including a systematic review find wound infections are detected earlier, readmissions and further surgeries are reduced, the burden on the hospital services is reduced, carbon emissions are reduced and patient satisfaction is high.^9^,^11^ Digital surgical wound monitoring is being used in several countries including the USA, the UK and Brazil, and its uptake is increasing. For example, at one group of hospitals in the UK, over 7000 surgical patients have submitted over 50 000 images for review over the past 3 years.^10^ This creates a new and additional workload for staff.

Artificial intelligence (AI) has been used to support the assessment of chronic wounds,^12^ but as yet, there has been limited use of AI in the monitoring of surgical wounds or the diagnosis or infections. One study was identified that used machine learning to predict patients that were likely to develop an SSI following orthopaedic surgery and another study in Spain used AI to identify patients with SSIs by reviewing patients’ clinical records.^13 14^

The aim of this feasibility study is to assess a surgical wound monitoring platform that uses AI to assist clinicians to review patients’ wounds by prioritising images for urgent review. This will manage staff time more effectively and expedite treatment.

The objectives are to obtain safety, feasibility and acceptability outcomes data plus obtain data for economic modelling.

## Methods and analysis

### Study design

This is a two-centre, unblinded parallel-group randomised feasibility study with safety, acceptability and health economic outcomes comparing the AI module with standard care. 120 adult patients having coronary artery bypass grafting (CABG) surgery will be randomised into two groups. Both patient groups will receive standard wound care following surgery, with the intervention group also receiving a digital wound monitoring platform with an AI module. Data are collected at baseline, and 30 days and 60 days after surgery through surveys, interviews, case note review, platform review and phone calls. The protocol was developed using the Standard Protocol Items: Recommendations for Interventional Trials guidelines.^15^

### Patient and public involvement

To determine patient and public views on using digital surgical wound images, we commissioned a national survey through the patient association which received around 400 completed responses.^16^ Our evaluation of the monitoring platform at five cardiac centres with 137 patients helped identify outcome measures, and 30 bedside pilots helped with platform development.^17 18^ This patient engagement led to changes to the platform relating to, for example, text message content, camera function and an email option. Our trial protocol was discussed with a Cardiovascular Lay Advisory Group. We have patient and public involvement (PPI) and equality, diversity and inclusion (EDI) representatives as coapplicants for the study and people with lived experience are represented on our Research Steering Group. This paper is coauthored with PPI and EDI representatives.

### Eligibility criteria

#### Settings

Two cardiac centres have been selected for recruitment: one in the north of England and one in the south. One city-based centre provides a wide range of patient ethnicities and performs around 1000 cardiac procedures each year. The second centre has a wide geographical spread including patients from rural and coastal locations and performs around 300 cardiac procedures each year.

#### Participants

Patients are eligible to take part if they are 18 years old, or over, having CABG surgery. Patients having first or redo CABG surgeries with or without adjunct cardiac procedures such as valve replacement, or chest reopening during the same admission as index surgery are eligible. Patients remain eligible despite the presence of a wound complication or infection, providing it is not a pre-existing SSI. Patients without a smartphone are eligible if they are willing to use a smartphone or internet provided by the study or if their next of kin or carer has a smartphone. Patients with a physical disability such as visual impairment will be eligible if their next of kin or carer is able to provide assistance.

Patients will not be eligible to take part if they require a ventricular assist device or extracorporeal membrane oxygenation, are ventilated or unconscious.

Patients who do not speak English and do not have a carer who can translate for them will not be able to participate in this feasibility study because of the need for free-text communication between patients and Isla. We are interested to know if/how age, sex, location (eg, rural/urban), socioeconomic status, ethnicity, English-speaking/non-English-speaking and carer involvement influence patient engagement with digital wound monitoring. However, we acknowledge that, due to the limited number of patients in this study and the restriction requiring English speakers (or patients with English speaking carers) any findings will be tentative and will require confirmation in a later definitive study.

#### Sample size

This is a feasibility study, and therefore, sample size calculations cannot be calculated as there are no current data which can be used as the basis of the calculations. The feasibility outcomes for this study are all binary outcomes, for which the widest CIs will occur with a proportion of 0.5 (or 50%), therefore, calculating a sample size for the CI based on 50% gives a ‘worst case’ estimate.

Recruiting 60 patients for each treatment group into the trial (ie, a total of 120 patients) will enable us to estimate the recruitment and drop-out rates with a 95% CI of within ±10% even if the rates were 50% for these two feasibility outcomes. (The 95% CI for a sample size of 120 patients and 60 successes, ie, 50% of patients recruited, or 50% of patients completing the study, is 41% to 59%). If the adherence to treatment rate is 50%, the adherence rate and 95% CI could be estimated to within ±13%. (The 95% CI for 30 adherent patients out of 60, as this is only relevant for patients in the intervention group, is 37%–63%). The sample size estimations were calculated using STATA V.18 with command ‘cii proportions’.

In addition, national data show a wound problem rate of 21% (our data shows 15%) with a cardiac wound infection rate of 8%.^19 20^ A sample size of 120 patients should provide around nine patients in each group with a wound problem of which 4–5 patients should have a wound infection. This is sufficient to inform feasibility, safety and acceptability data.

With a potential sample size of 1300 patients, we plan to recruit 120 patients over 10 months, a recruitment rate of around 11%. This should be achievable as a randomised trial using patients’ smartphones to monitor surgical wounds had a 69% recruitment rate.^9^

### Study procedure overview

**Figure 1:**
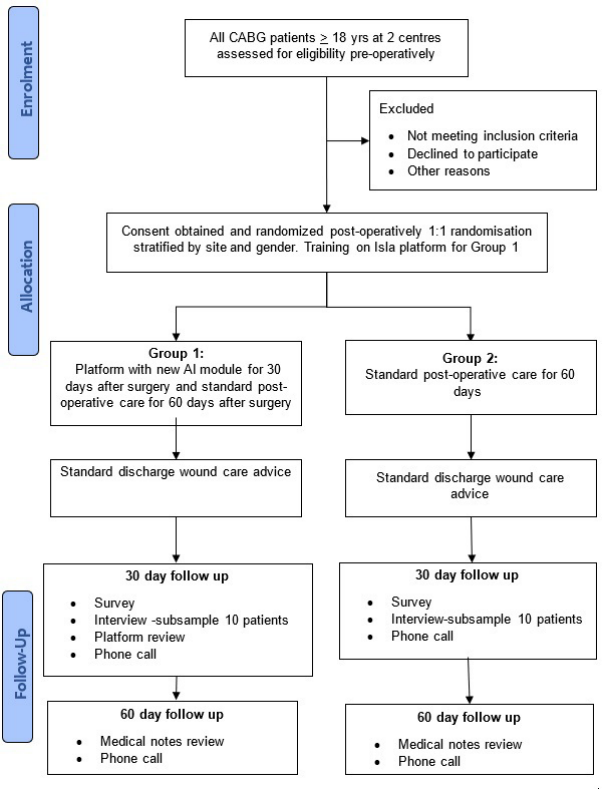
WISDOM Study flow diagram. AI, artificial intelligence; CABG, coronary artery bypass graft; WISDOM, Wound Imaging Software Digital platfOrM.

Each participant will be involved for 2 months (baseline to 60-day follow-up). Patients will be enrolled on the study and randomised after surgery but prior to discharge or up until day five after surgery, whichever comes first. Patients in the control group will have standard postoperative wound care follow-up. Patients in the intervention group will receive digital surgical wound monitoring with the AI module for 30 days after surgery and also standard postoperative wound care follow-up. 30 days after surgery, all patients will be invited to complete an online survey, and a subsample of ten participants from each group will be interviewed. All patients will be phoned at 30 days and 60 days. The participant’s journey through the study is shown in the flow diagram in figure 1. Participants are free to withdraw at any time without providing a reason and without affecting their ongoing care. Additionally, staff will be invited to complete an online survey after 30 days and a subsample of 10 staff will be interviewed.

### Recruitment and consent

Initial screening by a member of the usual care team will take place before surgery with final eligibility for study enrolment assessed after surgery.

Potential participants will be identified by the usual care team through screening surgical admissions lists and will be given brief information about the study at admission and when they are in the postoperative ward after surgery. If they express an interest in taking part, they will be provided with a leaflet and an opportunity to discuss the study and ask questions. This will be done by an appropriately delegated member of the team. The information will make it clear that the decision for whether they are allocated digital monitoring with the AI module, or not, will be random.

For patients admitted preoperatively, information will be provided preoperatively giving a minimum of 24 hours between receiving information about the study and seeking consent. For patients transferred in on the day of surgery, information will be given postoperatively with a minimum of 24 hours before consent is sought.

Informed consent will be obtained before any trial activities commence including data collection. Participants will be asked at enrolment how they wish to receive dissemination information about the study (text/email/post). Participants who complete data collection will be offered financial recompense for their time. We will not recruit patients who are unable to give consent.

As the interviews will be remote, consent for patients participating in the substudy will be conducted remotely. The researcher will cover the information leaflet and consent form with the participant on the recording, but only the researcher will sign the consent form. The participant giving consent will be audio recorded and transcribed.

Participants are free to decline involvement in the study without giving a reason, although where reasons are given, these will be collected as part of screening data. If patients do not wish to participate, they will continue to receive standard postoperative care and their standard of care will not change.

### Randomisation and allocation

Participants will be assigned to the intervention or control group using stratified 1:1 randomisation with mixed block sizes to maximise the chances of equal allocation to groups. The participants will be stratified by hospital site and sex (male, female, intersex). Participants will not be blinded as to their group allocation. This is because of obvious differences between the intervention and standard care. The hospital staff who are delivering wound care will be aware of the patient’s allocation status. Emergency unblinding will not be required as the study will not be blinded. Randomisation will take place at enrolment.

Randomisation will be implemented in the electronic data capture system used by the clinical trials unit. The sequence generation will be based on a random seed, selected by the study statistician, who will have no prior knowledge of the allocation sequence until after generation.

Participants will be allocated a treatment arm in the electronic case report form, via a randomisation form, where allocation details will be stored online and an email notification sent to the study team. Access to the online randomisation system will be via personal username and password, and specific to role.

If participants are no longer eligible for randomisation, they will be considered ‘consented, but not randomised’ and will not contribute to the required sample size.

### Intervention

#### Standard care group

Participants will receive standard surgical wound care follow-up, but not the digital wound monitoring with AI module. Standard wound follow-up after cardiac surgery may include an outpatient visit or telemedicine appointment 4–6 weeks after surgery, advice to contact the general practitioner (GP) or the hospital if the patient has concerns, and a postdischarge SSI surveillance questionnaire through the post or over the phone at 30 days.

### Digital wound monitoring with AI module group

Participants in the intervention group will receive the digital wound monitoring with an AI module in addition to standard surgical wound care follow-up. Participants will not be aware of AI output. Only clinicians interact with the AI component.

Isla is a browser-based digital platform which is used to monitor patients’ surgical wounds by collecting self-reported data (wound images and questionnaire responses) in response to preprogrammed regular short message service (SMS) text or email messages. Patients can use Isla on any modern phone, tablet or computer, there is no App to instal or download. Staff access is through existing tablets used in hospitals. The data are reviewed by clinicians, and patients are informed that there are no wound healing concerns or are contacted to obtain additional information or prescribe treatment.

The component under investigation in this study is a new module for the Isla platform which uses AI to identify signs of non-healing on wound images. Non-healing signs are redness or discolouration, wound gaping, unexpected tissue or fluid, or removable stitches or surgical clips detected more than 14 days after surgery. While clinicians will review all images, the images identified by the AI platform will be put forward for urgent priority review by Isla software. The new wound prioritisation module is being validated for predictivity, sensitivity and specificity, and inter-rater reliability.

Patients will receive verbal and written information on how to respond to SMS text messages and email links. Patients in the intervention group will be contacted via SMS text message, or email if preferred, 7 days, 14 days and 21 days after surgery with the link request remaining open for 6 days until the next request is sent out. For each request, patients are asked to submit a photo of their wound and complete the UK Health Security Agency (UKHSA) wound surveillance questionnaire.^21^

### Outcome measures

#### Safety outcomes

The primary safety outcome is the quality of images received, assessed by clinicians at 30 days. A quality image is one that can be used to make a clinical decision.

#### Acceptability outcomes

Primary acceptability outcomes are clinician and patient satisfaction using surveys and interviews at 30 days, acceptability of the intervention including attitude, burden, perceived effectiveness, ethicality, understanding, opportunity costs and self-efficacy. Acceptability data will also include the acceptability of being involved as a study participant. Secondary outcomes, and more detailed reasons for compliance/non-compliance with digital monitoring, will be explored in the patient intervention group surveys and interviews, and the staff surveys and interviews.

#### Feasibility outcomes

Primary feasibility outcomes are the recruitment rate, adherence with the module (defined as submission of one photo during the 30-day period), loss to follow-up and reasons for loss to follow-up. Secondary outcomes are access/barriers to participation and willingness of participants to be randomised, attrition rates, suitability of assessment procedures and outcome measures (including time and resources required to conduct assessments), number and severity of wound problems/infections, date of diagnosis, wound-related hospital readmission, further surgery to treat wounds, prescribed wound treatments, prescribed antibiotics, clinic visits and GP visits.

#### Health economic outcomes

Primary health economic outcomes include the number and severity of wound problems/infections, wound-related hospital readmissions, time to review images, further surgery to treat wounds, prescribed wound treatments, prescribed antibiotics, clinic visits, GP visits, patient travel time and quality-of-life data (SF-6Dv2).

#### Other secondary outcomes

Additional data collected are the number of photos received per patient, number of wound images/non-wound images, number of quality images, number of requests for images that are complied with, number photos initiated by patients, number of follow-up requests and number of wound images correctly prioritised.

### Data collection

Data collected at 30 days after surgery will be through an online survey, online interviews for a subset of participants, questionnaire-based phone calls and a review of the Isla platform data. 30 days has been chosen as the data collection point as this is the recommended follow-up time for a wound infection as stipulated by the national wound surveillance programme run by the UKHSA.^20^ Further data are collected at 60 days to estimate quality of life and identify events such as hospital readmissions or further surgery which will be used in the health economic analysis. Data collected at 60 days after surgery will be through questionnaire-based phone calls and medical case note review.

Online surveys will be hosted on Dacima. The patient survey will focus on satisfaction with wound follow-up (and acceptability of the platform for intervention group participants only). Staff surveys will focus on satisfaction with wound monitoring including the Isla platform with AI. The survey is expected to take 10 min to complete. Interviews will be conducted via Teams, recorded using digital audio recording and transcribed. The focus of the patients’ interviews will be acceptability of the Isla platform, satisfaction with postoperative wound follow-up and acceptability of taking part in the study. Interviews are expected to last around 40 min. The Isla platform review will be conducted to collect data on the number of images submitted, dates of submissions and discrepancies between Isla wound prioritisation and clinician prioritisation. Phone calls at 30 days will include the UKHSA postdischarge SSI questionnaire to diagnose wound infection status, questions focusing on engagement with National Health Service (NHS) services (including treatment, antibiotics, readmissions, further surgery, GP or hospital visits) and a quality-of-life tool (SF-6Dv2). The phone call at 60 days will focus on engagement with NHS services and will include the SF-6Dv2 quality-of-life tool. Medical case note review will capture demographic data plus readmissions or further surgery.

### Data analysis

Descriptive statistics will be presented to summarise the distribution of baseline variables across each of the randomisation groups. Continuous variables (age, height, weight, body mass index, quality of life) will be reported with means and 95% CI, if shown to be normally distributed, using a combined skewness and kurtosis test, otherwise will be reported with medians and IQR. The categorical and ordinal variables (eg, sex, ethnicity, disability, diabetes, smoker status, skin tone, socioeconomic status, surgical procedure, length of stay, carer status, partner status, owned smartphone, resources given to patient such as ipad, mobile, pay as you go and internet) will be reported with frequencies and percentages.

Safety outcomes, such as the quality of images received and assessed by clinicians at 30 days, will be reported using frequencies and percentages. Qualitative data from the interviews will be analysed using thematic analysis.

For acceptability outcomes, variables that are continuous will be reported with means and CIs, if shown to be normally distributed, using a normality plot, otherwise, they will be reported with medians and IQRs. Categorical variables (eg, sex, ethnicity) will be reported with frequencies and percentages.

Feasibility outcomes relating to adherence will be reported as the number and percentage of adherent patients in the intervention group. To be adherent a patient needs to submit one photo with a completed questionnaire within the 30-day period. This will be compared with the progression criteria defined below (table 1). The definitive study will proceed (or not) based on these outcomes. The number and percentage of eligible patients recruited for the study will be reported. The feasibility outcomes will be reported with 95% CI.

**Table 1:** Progression criteria

Criteria	Do not proceed	Proceed with changes	Proceed
Recruitment	<10% of eligible patients consent	Between 10% and <25% of eligible patients consent	At least 25% of eligible patients consent
Adherence with the module	<40% of intervention patients submit one or more images	Between 40% and <80% of intervention patients submit one or more images	At least 80% of intervention patients submit one or more images
Loss to follow-up	<10% of intervention patients complete the study	Between 10% and <60% of intervention patients complete the study	At least 60% of intervention patients complete the study

For all secondary outcomes, continuous variables will be reported using descriptive methods such as means and 95% CI. Categorical variables will be reported with frequencies and percentages.

There will be no subgroup analysis, no adjusted analysis and no interim analysis.

### Staff study

All nurses and surgeons at both sites who have been involved in using the Isla platform will be invited by the site research team to complete an online survey. The survey is hosted on Dacima and will take 15 min to complete. The focus of the staff survey is the experience of being involved in the study, experience of using the Isla platform, and experience and views on postoperative surgical wound follow-up. Quantitative survey data will be analysed using simple descriptive statistics. Staff will be invited, via the survey, to participate in an interview. Up to 10 staff will be interviewed.

As the interviews will be remote, consent will be conducted remotely. The researcher will cover the information leaflet and consent form with the participant on the recording, but only the researcher will sign the consent form. The recording of the participant giving consent will be audio recorded and transcribed.

The interviews will be semistructured and focus on the acceptability of the Isla platform and satisfaction with postoperative wound follow-up. All interviews will take place via Microsoft Teams and will be digitally recorded with the permission of the participant. Interviews are expected to last around 40 min. Data from the interviews will be independently transcribed and analysed using thematic content analysis.^22^

### Health economic evaluation

An economic investigation is included as the provision of the intervention will involve additional upfront direct costs. The intervention is unlikely to be widely adopted unless it can be shown to be cost-effective. For the intervention to be cost-effective, the initial upfront cost will need to be offset by savings later in the clinical pathway and, or, deliver positive impacts on health outcomes. The economic investigation will comprise two stages: an initial scoping exercise and an economic evaluation on completion of the clinical study. The scoping exercise has been undertaken to inform the design of this study. This involved a search for relevant economic evaluations and existing evidence base, the development of an early model and the drafting of a health economic analysis plan.

The economic evaluation will compare health outcomes and costs in the intervention and control arms using a probabilistic decision model. This will provide an estimate of the probability of cost-effectiveness of the intervention compared with the standard of care and identify key parameters for cost-effectiveness for further investigation in a clinical trial. We will also explore the impact of different models of providing the intervention service.

### Implementation and adoption strategy

A healthcare adoption plan will be developed which will include stakeholder analysis, identification of potential barriers and facilitators to adoption, and the best route to the NHS market. We will map inclusion of the Wound Imaging Software Digital platfOrM solution in current NHS pathways and processes and maximise our early insight into the evaluation and adoption processes. We will collect the appropriate information during the project, with a view to understanding pathway changes and effect of health economics and end-user requirements on potential adoption.

### Ethics and dissemination

Permission to conduct the study was granted by the North of Scotland Research Ethics Committee 1 (24/NS0005) and the MHRA (CI/2024/0004/GB). The trial was prospectively registered on 15 March 2024 (ISRCTN16900119) with data collection starting in July 2024.

Data arising from the study will be owned by the sponsor and shared with collaborators in accordance with the Terms and Conditions of the study Collaboration Agreement. The study reporting will be in accordance with the Consolidated Standards of Reporting Trials guidelines.^23^ Our PPI group will inform the dissemination strategy. Study participants will be notified of publications via the study newsletters and via email if they choose to provide contact details. Study results will also be shared with stakeholders, identified through a stakeholder mapping exercise. Two publications are planned, which will be published in open-access journals. We will also share a study report and findings on the study website (www.wisdomai.uk) and through social media such as X, formerly Twitter accounts. Anonymised data will be available after study completion on request for research purposes. The clinical investigation will be registered on a publicly accessible database and the results of the investigation will be made publicly available.
