# Supplementary material for: Digital wound monitoring with artificial intelligence to prioritise surgical wounds in cardiac surgery patients for priority or standard review: protocol for a randomised feasibility trial (WISDOM)
Source: BMJ Open. 2024 Sep 17;14(9):e086486. doi: 10.1136/bmjopen-2024-086486 (PMC11409336; doi:10.1136/bmjopen-2024-086486)
Supplement: Supplementary file 1 [file bmjopen-2024-086486.reviewer_comments.pdf]

PEER REVIEW HISTORY

BMJ Open publishes all reviews undertaken for accepted manuscripts. Reviewers are asked to complete a checklist review form and are provided with free text boxes to elaborate on their assessment. These free text comments are reproduced below.

ARTICLE DETAILS

**TITLE (PROVISIONAL)**	Digital wound monitoring with artificial intelligence to prioritise surgical wounds in cardiac surgery patients for priority or standard review: protocol for a randomised feasibility trial (WISDOM)
**AUTHORS**	Tanner, Judith; Rochon, Melissa; Harris, Roy; Beckhelling, Jacqueline; Jurkiewicz, James; Mason, Lara; Bouttell, Janet; Bolton, Sarah; Dummer, Jon; Wilson, Keith; Dhoonmoon, Luxmi; Cariaga, Karen

VERSION 1 - REVIEW

**REVIEWER NAME**	***Lei, Mingxing***
**REVIEWER AFFILIATION**	General Hospital of People's Liberation Army
**REVIEWER CONFLICT OF INTEREST**	None
**DATE REVIEW RETURNED**	General Hospital of People's Liberation Army

**GENERAL COMMENTS**	This study aims to assess a surgical wound monitoring platform that utilizes artificial intelligence to assist clinicians in reviewing patients' wound images by prioritizing concerning images for urgent review. The main findings of the study will focus on the effectiveness of the artificial intelligence module in managing staff time more effectively, improving patient outcomes by detecting wound complications early, and assessing safety, acceptability, feasibility, and health economic endpoints.<br>The findings of this study will provide valuable insights into the feasibility and effectiveness of using artificial intelligence in surgical wound monitoring, potentially leading to improved patient care practices in the future. This study also provides a detailed overview of the research methodology, including the study design, sample size, allocation process, and outcome measures. By including such comprehensive details, this study ensures transparency and credibility in its findings and conclusions.<br>1. Consider incorporating patient feedback and perspectives throughout the study process to ensure that the artificial intelligence module is user-friendly and acceptable to patients.<br>2. Collaborate with healthcare providers and stakeholders to explore potential integration of the artificial intelligence module into existing healthcare systems for seamless implementation and adoption.<br>3. Conduct cost-effectiveness analyses to determine the economic benefits of using the artificial intelligence module in surgical wound monitoring, considering factors such as staff time savings and improved patient outcomes.

VERSION 1 – AUTHOR RESPONSE

REVIEWER COMMENTS
1 Consider incorporating patient feedback and perspectives throughout the study process to ensure that the artificial intelligence module is user-friendly and acceptable to patients
Response: Thank you for your suggestion, but it will not be possible to incorporate changes to the AI platform during the study as the lag time between collecting and analysing sufficient amounts of data, and making and testing AI platform changes would take too long to be implemented within the study. We have already carried out extensive evaluation and testing with patients using the platform (described in the 'PPI' section- Page 4 lines 32-46) and the new AI component does not affect the patient-facing aspect of the existing platform.
2 Collaborate with healthcare providers and stakeholders to explore potential integration of the artificial intelligence module into existing healthcare systems for seamless implementation and adoption.
Response: We have added our plans for implementation and adoption as requested into a new section headed 'Implementation and adoption strategy' Page 11 lines 55 - Page 12 line 7.
3 Conduct cost-effectiveness analyses to determine the economic benefits of using the artificial intelligence module in surgical wound monitoring, considering factors such as staff time savings and improved patient outcomes.
Response: The economic investigations are already discussed in detail in the section headed 'Health economic evaluation' (Page 11 lines 32-53) and specific outcomes including staff time and patient outcomes are already listed in the 'Outcome measures' section Page 9 lines 12-18.

VERSION 2 – REVIEW

**REVIEWER NAME**	***Lei, Mingxing***
**REVIEWER AFFILIATION**	General Hospital of People's Liberation Army
**REVIEWER CONFLICT OF INTEREST**	None
**DATE REVIEW RETURNED**	29-Jul-2024

**GENERAL COMMENTS**	My concerns were properly addressed. No more questions. Thanks a lot!
